# Exploring the lived experiences of individuals living with Charcot neuro‐osteoarthropathy in Australia: A qualitative research study

**DOI:** 10.1002/jfa2.70037

**Published:** 2025-05-01

**Authors:** Nathan Mark Chapman, Peta Ellen Tehan

**Affiliations:** ^1^ Podiatry High Risk Foot Service Hunter New England Local Health District New Lambton New South Wales Australia; ^2^ School of Clinical Sciences Faculty of Medicine, Nursing and Health Monash University Clayton Victoria Australia

**Keywords:** Charcot neuro‐osteoarthropathy, diabetes‐related foot disease, lived experience, qualitative

## Abstract

**Aim:**

Charcot neuro‐osteoarthropathy (CNO) is a rare but serious inflammatory process in individuals with peripheral polyneuropathy leading to the development of fractures, dislocations and permanent foot deformity. Most commonly, CNO occurs in individuals with diabetes and the progression of significant foot deformity can predispose the individual to pressure‐related ulcerations and increased risk of lower limb amputation. The lived experience of the individual with CNO is largely unknown. This study aims to explore the lived experience of individuals affected by active CNO and CNO in remission in Australia.

**Methods:**

This was a qualitative study using semi‐structured interviews of a heterogeneous purposeful sample of individuals with CNO from a high‐risk foot service in Australia. Interview questions were related to the physical, financial, social and emotional impacts of CNO and were developed based on the validated SF‐36 outcome measure tool. A reflexive thematic analysis approach was used to analyse the dataset.

**Results:**

Fourteen participants were recruited including seven males, seven females with age ranging from 36 to 74 years. Four themes were derived: (1) A burden to family and caregivers and feeling isolated and alone. (2) A lack of certainty relating to outcomes leading to increased anxiety. (3) Adaptive health behaviour changes made as a result of the CNO diagnosis. and (4) Limited access to healthcare information and healthcare support systems.

**Conclusions:**

The burden of CNO extends beyond the physical limitations of living with a significant foot deformity and the associated increased risk for further limb threatening complications. Individuals with CNO report feelings of increased anxiety, a loss of independence and feel they are a burden to their family impacting on their individual roles at home, including the ability to provide financially. This should be considered when managing individuals with CNO, with more holistic approaches to care required. This research highlights that increased engagement with mental health support services, social work and peer‐group support along with greater access to evidence‐based information on the management of CNO may better support the psycho‐social needs of this population.

## 1 BACKGROUND

Charcot neuro‐osteoarthropathy (CNO) is a rare but serious limb‐threatening complication associated with peripheral polyneuropathy, most commonly occurring in people living with diabetes [1]. Charcot neuro‐osteoarthropathy is a progressive disease process which causes injury to the ligament and soft tissue and destruction of bone, often resulting in permanent foot deformity [2]. Charcot neuro‐osteoarthropathy is also frequently complicated by ulceration. When this occurs, individuals experience a 12 times higher risk of amputation compared to an individual with CNO alone [3]. The prevalence of CNO is considered largely unknown, with current international guidelines estimating that 0.3% of people living with diabetes have CNO with an additional 160,000 new cases diagnosed annually [1].

It is currently proposed that CNO occurs as a result of an initial trauma, which results in an acute inflammatory response in the foot or ankle of an individual with peripheral neuropathy [4]. As a result, pro‐inflammatory and anti‐inflammatory cytokines are released, resulting in an increase in osteoclast activity and subsequent resorption of bone [5]. Combined with repetitive force from mobilisation in an insensate foot, weakening of ligaments, joint dislocations and fractures occur [6], presenting clinically as a red, hot, swollen foot [7]. Management of CNO involves prolonged periods of immobilisation in knee‐high offloading devices until the active disease process has resolved [1]. This often has significant impacts on individuals’ ability to socialise, work and participate fully in society. Beyond the active stage, CNO in clinical remission requires long‐term care and review due to the increased risk of complications including ulceration from residual deformity, the increased risk of contralateral disease, and the reduction in long‐term survival as a result of cardiovascular morbidity and mortality [3, 8]. Much remains unknown about CNO, including the lived experience of individuals with the condition in both active and clinical remission stages. International quantitative studies have demonstrated substantial decreases in quality of life amongst individuals living with CNO [9, 10, 11]; however, these studies also clearly articulate the difficulties and limitations in utilising generic outcome measures that do not accurately capture the emotional distress experienced by patients with diabetes‐related foot disorders [9, 12]. Therefore, a qualitative approach, which allows for greater insights into the lived experience of individuals and how this influences behaviour, feelings and values, is needed [13].

The first study to utilise a qualitative approach, to gain a deeper understanding of lived experience of active CNO, was completed by Gooday et al. (2022) in a cohort of active CNO patients living in England. The study by Gooday et al. described how the consequences of CNO extend beyond physical and have substantial socio‐economic and psychological impacts. Individuals with CNO in this study commonly reported low mood and low self‐esteem, and recommendations were made for a more holistic approach in the care of individuals with CNO. Given this is the only qualitative study completed in people with CNO to date, and results are not transferrable, this area remains underexplored, and more research is needed to further elucidate how individuals with CNO negotiate their daily lives and relationships, seek support and determine their needs to improve management strategies. Furthermore, different populations with differing health systems and cultural values should also be further explored and understood. Understanding local populations is important to inform local models of care and support mechanisms.

Furthermore, given that the long‐term impact of CNO reaches well beyond the active phase, it is also important to explore the perceptions of people with CNO in remission. Therefore, the aims of this qualitative study are to explore the perceptions and lived experiences of Australian people with diabetes and active CNO and CNO in clinical remission. Furthermore, this study aimed to gain a deeper understanding of the impact of CNO on physical, social and emotional wellbeing, as well as broader impacts on the person as a whole.

## 2 METHODS

A qualitative study employing a reflexive thematic approach with semi‐structured conversational style interviews was undertaken to explore the perspectives and experiences of individuals with Charcot neuro‐osteoarthropathy relating to the physical, financial, social and emotional impacts of the condition. Participants were eligible if they were aged over 18 years with a diagnosis of type one or two diabetes and CNO confirmed by hospital records. Participants were not eligible if they had a cognitive disorder which affected their ability to provide consent or participate in interviews.

Participants were recruited from the high‐risk foot clinic at John Hunter Hospital in Newcastle, NSW, Australia. Ethical approval was provided by the Hunter New England Human Research Ethics Committee of Hunter New England Local Health District (HNELHD HREC 2022/ETH00992). Participants meeting the inclusion criteria attending the clinic were invited to participate via an invitation letter provided in person at prior clinical appointments. Potential participants were then asked to express interest, and a heterogenous sample was recruited including a mix of gender, diabetes type, age, CNO stage and duration and living situations. Informed written consent was obtained from all participants. Interviews were conducted between August 2022 and January 2023 in a private clinical room by a single researcher (NC), a qualified podiatrist with training in interview methods.

Basic demographic data were collected during interviews including participants gender, age, diabetes type, living situation and CNO stage and duration. Interview questions were developed by undergoing a literature review of research relating to patient's lived experiences and perspectives on CNO and the SF‐36 outcome measure tool. Ten questions were formulated, and an interview script was developed (Appendix 1). The first question, “Can you tell me about your foot problems?” was created with the aim of developing a rapport between the researcher and participant and forming a comfortable space for the participant to express their perspectives and experiences. Interviews were digitally recorded by the interviewer and transcribed verbatim to keep the interviews original in nature, which aligns with a reflexive thematic approach [14]. After the interview, participants were provided an opportunity to review their transcripts to reduce the risk of inaccuracies arising in the data.

Participants were recruited until researchers determined that data saturation was achieved. It was deemed that no further interviews would provide any new insights or experiences. The dataset was analysed by utilising the six phases of reflexive thematic analysis described by Braun and Clarke [14]. Two researchers familiarised themselves with the dataset through multiple readings (P.T. and N.C.). Through this familiarisation, patterns of meaning were identified, with latent and semantic codes generated, labelling important features of the data that are related to the research aim. Codes were then clustered, and broader patterns of meaning were developed, forming initial themes. The initial themes were then refined and compared to the dataset to ensure they reflected the true meaning of the data. Final themes were then identified and named [14].

## 3 RESULTS

A total of 14 participants were recruited, with interview times taking up to 42 min. Seven males and seven females were recruited, aged between 36 and 74 years of age (Table 1). None of the included participants had experienced a major amputation.

**TABLE 1:** Descriptive information relating to participants (attached).

Participant number	Sex	Age	Living situation	DM status	DM duration years	ISED quintile	Charcot stage/history
1	M	61	Lives with elderly father	T1DM	23	2	Bilateral Charcot in clinical remission
2	F	48	Lives alone	T1DM	33	3	Right foot active Charcot
3	F	69	Lives with husband	T2DM Insulin dependent	31	1	Right foot Charcot in clinical remission Charcot
4	M	58	Lives with wife	T2DM	20	4	Right foot Charcot in clinical remission
5	M	71	Lives with housemate	T2DM	25	1	Bilateral Charcot in clinical remission
6	F	36	Lives with family	T1DM	23	1	Right foot Charcot in clinical remission
7	F	52	Lives with husband	T2DM	17	2	Left foot active Charcot
8	F	48	Lives with husband/children	T1DM	29	4	Left foot Charcot in clinical remission
9	F	48	Lives with husband/children	T2DM	19	4	Left foot Charcot in clinical remission
10	M	56	Lives alone	T2DM	14	1	Bilateral Charcot in clinical remission
11	M	66	Lives with wife and older son	T2DM	23	3	Right foot Charcot in clinical remission and left foot active Charcot.
12	F	66	Lives with husband	T2DM	15	1	Right foot Charcot in clinical remission
13	M	51	Lives with wife	T2DM	12	3	Right foot Charcot in clinical remission
14	M	74	Lives with husband	T2DM	22	2	Left foot Charcot in clinical remission and right foot active Charcot.

Four themes were derived from the data set: A burden to family and caregivers and feeling isolated and alone, a lack of certainty relaing to outcomes leading to increased anxiety, adaptive health behaviours made as a result of the CNO diagnosis and limited access to healthcare information and healthcare support systems.

### 3.1 A burden to family and caregivers and feeling isolated and alone

This theme was generated in response to the participants’ perceptions of being a physical, emotional and financial burden to their family and caregivers which also influenced an individual feeling of loneliness and sense of stigma associated with CNO.

Many participants reported a negative impact on their roles and responsibilities in the home, resulting in increased dependency on family members for daily tasks. There was also a consciousness about the emotional strain on their caregivers. Many participants shared stories of family and caregivers needing to assist with activities of daily living, which was associated with a sense of embarrassment, particularly as most had never experienced any form of disability prior to the diagnosis of CNO. Furthermore, for many participants, a diagnosis of CNO was associated with an inability to work, which resulted in financial stress, and associated burden to their family.

When I wasn't allowed to walk on them, my wife had to do everything and yeah. I've had to use a toilet chair. She had to shower me…. I couldn't work because my job entails walking on my feet all day. So, they just cancelled that out. And then of course I didn't have any, uh, income insurance. I had to use all my long service, so I was still getting paid, but I was only just getting my long service. I used; I haven't got no long service left now.

(Participant 11)

I haven't worked since, haven't worked there. Cause I'm a truck driver so I can't, can't work

(Participant 4)

Feelings of isolation and loneliness were also associated with this reduction in independence as a result of a loss of mobility, inability to drive and the use of either a non‐removable or removable offloading device. This loss of independence resulted in reduced participation in social and community activities; Perceptions of judgement and feelings of loneliness were commonly discussed, as well as being misunderstood due to a lack of CNO awareness.

I can't go anywhere. I can't walk. Um, my friends don't really come and see me. My mental health has declined because I'm having bad panic attacks…….I can't go get my haircut because of the wheelchair. I can't go to the movies because of the wheelchair. So we are finding it hard to go places we usually go because of the wheelchair.

(Participant 7)

I got all my mates that asked me why I was still wearing a boot that I can't, I just tell 'em I've got the Charcot in it, I can't explain why, you know

(Participant 5)

### 3.2 A lack of certainty relating to outcomes leading to increased anxiety

This theme captures the regular reporting of increased health‐related anxiety of participants. This is related to progression of the disease process, fear and uncertainty about long‐term outcomes, including a fear of amputation. Exacerbation of existing anxiety in relation to their general health and management of chronic conditions was commonly reported. There was also common reporting of feelings of self‐consciousness and worry relating to how others perceived them. The lack of certainty relating to the treatment duration was also proposed to be a major cause of anxiety.

Charcot exacerbated everything that I had going on before, like my diabetes, um, just with stress levels and anxiety

(Participant 2)

I’m still on antidepressant tablet to this day because of it. And I'm trying to get off them, but they're not the easiest thing to get off

(Participant 1)

I was more concerned about that, you know, I was going to be, um, bedridden or I was going to lose my feet

(Participant 8)

### 3.3 Adaptive Health Behaviour Changes made as a result of the CNO diagnosis

This theme captures the lifestyle and behavioural changes that participants made as a result of being diagnosed with CNO. The adaptive adjustments in behaviour were reported as both positive and negative. Some participants discussed taking greater control of their diabetes and lifestyle factors, post diagnosis of CNO. For many others, however, there were maladaptive changes in adherence to treatment such as offloading. These were further associated with onward effects into their social and emotional wellbeing.

I have got my blood sugars down a bit, like my blood sugars were probably a bit high back then as well.

(Participant 12)

I put on 30 kilos after I was diagnosed and being in the full contact cast

(Participant 2)

Because the first time that I got Charcot Foot in 2018, I, that day, I said, I quit drinking. I haven't drunk since

(Participant 11)

So, I've done things that I shouldn't have done, but it was more of, I don't know, fighting back to the system. Yeah. Well, you can't tell me I can do that. I can, I can do it. but then it had repercussions that maybe if I didn't do what I'd done, maybe I might've been off my feet at a shorter period of time

(Participant 13)

### 3.4 Limited access to healthcare information and healthcare support systems

This theme relates to accessing support from healthcare professionals and accessing accurate and timely information related to CNO. Many participants conducted their own research on their disease, mainly from a range of internet sources, including online social networking sources. Information was perceived as scarce or unreliable most of the time. Therefore, there was an increased reliance towards health professionals in seeking reliable information. This was associated with both positive and negative experiences for participants. Commonly, participants expressed generally a poor awareness of the condition among health professionals involved in their care. This, many times, led to delays in diagnosis, incorrect or delayed management, which resulted in a general lack of trust and confidence in many healthcare providers.

I probably would be less inclined to see a podiatrist outside of the hospital, especially a podiatrist that didn't know about Charcot

(Participant 2)

I sort of went to try and look it up about it, but it, it doesn't actually explain how it happens as like all the time. You know, like I suppose some of the information's a bit scarce

(Participant 12)

There's not a lot of information kind of, I don't know, it's more so America and I joined the Facebook group on there

(Participant 2)

## 4 DISCUSSION

This was a qualitative research study exploring the perceptions and experiences of people with CNO. To the best of the author's knowledge, the current study is one of only two qualitative studies exploring lived experience in CNO to ever have been completed, so adds further value to the knowledge base. Four themes were derived from the data: a burden to family and caregivers and feeling isolated and alone, a lack of certainty relaing to outcomes leading to increased anxiety, adaptive health behaviours made as a result of the CNO diagnosis and limited access to healthcare information and healthcare support systems. These themes capture the unique challenges faced by people with CNO and give deeper insights into their lived experiences. The findings of the current study complement the qualitative work by Gooday et al. (2022) in a Glasgow cohort of CNO, demonstrating similarities yet also distinctions.

The first theme in the current study‐ “A burden to family and caregivers and feeling isolated and alone;” relates to isolation and loneliness, driven primarily due to the loss of independence, reduced mobility and feeling of social burden and stigma associated with their CNO diagnosis. This theme is similar to Gooday's “trapped at home isolated and missing social life and daily routines” [15]. Feeling isolated has considerable impact on a person’s involvement in social activities and day‐to‐day interactions [16]. This feeling of alienation or loneliness can have further detrimental health impacts on participants due to the bidirectional relationship between poor health and social isolation [17, 18]. It is well reported in the literature that social relationships and social supports can improve physical and psychological wellbeing, indirectly and directly [19]. There are several proposed mechanisms for this, one of which is people should feel a sense of meaning in their lives or have a role‐based purpose and feel a sense of mattering [19]—all of which were reported to be negatively impacted by participants in the current study as a result of their CNO diagnosis. Many participants discussed limitations in being able to fulfil their own roles and responsibilities in the home or community, with an increased reliance on family and friends leading to a perceived sense of social burden.

The theme, “A lack of certainty relating to outcomes leading to increased anxiety” was developed to highlight the common reporting of these feelings from people with CNO, which underpinned much of the participants’ dialogue and subsequent behaviours. For some participants, the diagnosis of CNO exacerbated underlying mental health conditions. For others, however, the rigid treatment regimen and subsequent loss of independence resulted in increased anxiety levels, some of which stemmed from uncertainty and an apparent loss of control over their health outcomes. Uncertainty about outcomes is understandable given that the duration of treatment and the need for immobilisation in CNO can vary significantly and be unpredictable. A recent systematic review reported that CNO treatment time varied between 9 to 12 months before remission, with up to 2 years of treatment reported in other studies [20, 21]. High levels of anxiety have also previously been reported in people with CNO, with females, ethnic minorities and people out of work reported to have even greater levels of anxiety [22]. Likewise, a recent meta‐analysis demonstrated that up to 40% of patients with type 1 or 2 diabetes are living with anxiety [23]. Furthermore, mental health disorders and diabetes have further been associated with increased non‐adherence to medical treatment, decreased quality of life and increased mortality [24]. This theme also confirms the findings of a recent multiple methods study completed within the current study's health service area, which also highlighted the negative impact of diabetes‐related foot ulceration on mental health and wellbeing in people with diabetes‐related foot disease [25].

The “Adaptive Health Behaviour Changes made as a result of the CNO diagnosis” theme was developed as many participants discussed changes in their behaviour as a result of their CNO diagnosis. There was a divergence between some reporting making positive behaviour changes as a result of their diagnosis, including taking greater control of their blood glucose control amongst other positive changes. For others, however, there were also maladaptive behaviours and unintended consequences, often as a result of the treatment regimen, particularly non‐adherence to strict offloading protocols. Supporting people with CNO in making and sustaining positive behaviour change is an integral part of effective care [26], and a patient's motivation for behaviour change has been identified as a predictor of health outcomes [27]. The way in which health professionals interact with people with CNO can significantly influence the individuals’ level of motivation to make behaviour change [28]. Motivational interviewing aims to increase the patient's intrinsic motivation to make and sustain changes in behaviour [29]. Although there is limited evidence to demonstrate the effectiveness of this technique in diabetes‐related foot complications [29], there is evidence in other conditions and settings including primary care [30, 31]. This therefore may be an important tool in delivering person‐centred care in people with CNO and could potentially play a role in improved adherence with treatment regimens. Difficulty with behaviour change and poor adjustment and coping with chronic disease has been associated with a sense of feeling stigmatised [32] and further contributes to poor mental health which can directly interfere with treatment adherence [33]. The wearing of suitable offloading is an overt sign of the CNO disease process which may induce a sense of stigma in people with CNO, with some of the participants discussing this within their interviews.

The final theme, “Limited access to healthcare information and Healthcare”, highlights that many participants felt there was inadequate support for people with CNO. There was difficulty in finding trusted information, a distinct lack of peer support and a lack of awareness amongst health professionals of CNO. The lack of awareness for many resulted in a delay in initial diagnosis. Delayed or misdiagnosis in CNO is common, with previous research reporting that up to 79% of cases, confirmation of a CNO diagnosis can be delayed up to 29 weeks [34, 35]. Many of the participants relied heavily on carers and family members to provide active coping assistance, which led some participants to believe they were well supported. Peer support through experienced peers has been demonstrated to be an effective method to provide support to people living with chronic conditions [36]. This has been proven effective in wound care, with the development of Lindsay Leg Clubs [37] for leg ulcer patients to connect in safe spaces facilitated by a health professional who can answer clinical questions whilst connecting patients to each other to provide peer support and sharing of experiences. Given the prolonged treatment period for individuals with CNO, greater holistic support of individuals with CNO is needed, and peer support would be one potentially effective mechanism to deliver this support. Although multi‐disciplinary care is universally accepted as best practice in the treatment of diabetes and foot complications, psychology and social work are often not included as part of high‐risk foot services. These health professionals can assist with many of the issues within the themes derived in the current study, such as anxiety, and have the ability to make significant contributions to holistic care. This increased involvement may result in increased adherence to treatment protocols which may, in turn, result in better long‐term outcomes.

Future CNO research should build upon the results of the current study to inform an evidence‐based resource to provide better ongoing management and holistic support to people with CNO. The health practitioners' perspectives should also be captured. Further exploration of lived experience should also be completed in other countries with differing health systems, including developing countries.

## 5 LIMITATIONS

Although we recruited a heterogenous sample of participants that were reflective of an Australian population with acute CNO and CNO in clinical remission, these results may not be transferable to the broader population with CNO. We only included participants with diabetes, so the experience of individuals without diabetes and CNO may not be comparable. Both researchers were clinical podiatrists, so they approached the data with this implicit bias. The researchers’ roles as podiatrists may also have influenced the responses of the participants. Participants may have focused more on physical and foot‐related issues and complaints rather than broader social and mental health issues.

## 6 CONCLUSIONS

Charcot neuro‐osteoarthropathy is an uncommon, complex and devastating disease process. Charcot neuro‐osteoarthropathy can not only lead to physical deformity and amputation but, as the findings of this study demonstrate, can predispose individuals with CNO to increased feelings of anxiety and isolation, loss of independence as well as changes to their personal roles in the home and a reliance on friends and family. Increased health professional awareness of these social challenges faced by individuals with CNO is needed in order for health professionals to connect individuals with services such as psychology and social work. Difficulties in accessing available and trustworthy resources in addition to the perceived lack of awareness of CNO among health professions had further attributed to participants reliance on carers and family members. Evidence‐based patient education resources and facilitating experiential peer groups would be likely welcomed in individuals with CNO as trusted, valuable sources of information and support. This increased support may assist in achieving better overall health outcomes and quality of life in individuals with CNO and their families.

## AUTHOR CONTRIBUTIONS

**Nathan Mark Chapman:** Data curation; formal analysis; resources and writing original draft. **Peta Ellen Tehan:** Conceptualisation; formal analysis; methodology; administration; supervision; writing review and editing.

## CONFLICT OF INTEREST STATEMENT

The authors declare no conflicts of interest.

## ETHICS STATEMENT

Ethical approval provided by the Hunter New England Human Research Ethics Committee of Hunter New England Local Health District (HNELHD HREC 2022/ETH00992).

## TRIAL REGISTRATION NUMBER

Not applicable.

## Data Availability

The data that support the findings of this study are available on request from the corresponding author. The data are not publicly available due to privacy or ethical restrictions.

## APPENDIX 1 CHARCOT NEUROARTHROPATHY IMPACT ON QUALITY OF LIFE INTERVIEW GUIDE

### A1 Introduction

Can you tell me about your foot problems?

Are you aware of your Charcot diagnosis and what this means?

When and how was your Charcot diagnosed?

### A2 General health and wellbeing

How do you currently consider your general health in comparison to before your Charcot diagnosis?

### A3 Health literacy

Do you consider yourself to have a good, average or poor understanding about your Charcot foot? This includes the disease process, treatment aim and risks.

### A4 Physical Health

Has your Charcot foot diagnosis impacted on your physical health, mobility or independence?

### A5 Financial impacts

Since learning of your diagnosis of Charcot, has your ability to work or financial situation changed?

### A6 Social impacts

Has your Charcot foot diagnosis interfered with your relationships and/or social activities?

### A7 Emotional wellbeing

Since your diagnosis of Charcot, have you had any of the following problems with your work or other regular daily activities as a result of any emotional problems (such as feeling depressed or anxious)?

### A8 Podiatry involvement

How do you feel about attending regular podiatry appointments to assist in the management of your Charcot foot? Do you consider attending these appointments to be a positive or negative experience?

### A9 Support services

During your experience with Charcot, have you sought the support from services of a health professional (psychologist, social worker, and GP)? Have you ever been provided with information about available support services?

### A10 Reflection

Reflecting on your experience with Charcot, is there anything which you think may help someone else navigate the process? For example, support groups, referral to mental health professionals and more resources to learn about Charcot.
